# Long term monitoring of the reproductive behavior of wild Chinese pangolin (*Manis pentadactyla*)

**DOI:** 10.1038/s41598-021-97618-4

**Published:** 2021-09-13

**Authors:** Nick Ching-Min Sun, Kurtis Jai-Chyi Pei, Li-Yue Wu

**Affiliations:** 1 Graduate Institute of Bioresources, College of Agriculture, National Pingtung University of Science and Technology, Pingtung, Taiwan; 2 Department of Entomology, National Chung Hsing University, Taichung, Taiwan; 3 IUCN SSC Pangolin Specialist Group, Zoological Society of London, London, UK; 4 Institute of Wildlife Conservation, College of Veterinary Medicine, National Pingtung University of Science and Technology, Pingtung, Taiwan; 5 Pingtung Rescue Center for Endangered Wild Animals, National Pingtung University of Science and Technology, Pingtung, Taiwan

**Keywords:** Ecology, Zoology

## Abstract

Observations of Chinese pangolin (*Manis pentadactyla*) in the wild are extremely rare and challenging because of their nocturnal and cryptic activity patterns and low population density. The present article reported the first field observation in eastern Taiwan, from October 4, 2012 to June 16, 2016, on the reproductive behavior of the Chinese pangolin based on the monitoring of a female (LF28) using radiotelemetry and camera traps. During this period, LF28 aged from 1–4.5 years old and gave two single-births, both took place in early December, at 3 and 4 years old, respectively. We recorded the entire 157 days of the first nursing period from parturition to maternal separation. For the second infant, the gestation period was estimated to be around 150 days based on the evidence that the pregnancy started in early Jul. 2015 and the offspring was born on Dec. 9, 2015. During the entire nursing period, LF28 frequently moved the offspring from one nursing burrow to another staying various durations ranging from 1 day to more than 35 days, and almost all (= 15/16) of these burrows were located in the core (MCP75) of LF28’s home range. Started from the month of parturition and lasting throughout the whole nursing period, different adult males constantly visiting the nursing burrows were recorded. Mating behavior was recorded once outside the burrow in March, which provided evidence of the occurrence of post-partum estrus in this species. Delayed implantation was proposed based on the observation of a several months lag between copulation and the estimated pregnancy initiation date. The present study demonstrated the advantage of using remote technologies to learn the life history of resting fossorial species.

## Introduction

Field observations of the Chinese pangolin (*Manis pentadactyla*) are extremely rare and difficult due to their nocturnal and elusive behavioral patterns^1^, as well as very low population size in the wild^2^. Pangolins are fossorial and frequently use their powerful forelimbs to excavate ground burrows not only to search for ants or termites (i.e., the foraging burrows), but also to create shelters used for resting, giving birth and nursing offspring (i.e., the resting or nursing burrows)^3,4^.

It has been estimated that, in eastern Taiwan, the pangolin burrow density can be as high as 110/ha in a habitat with a density of 12.8 pangolins per 100 ha^5^. However, very few (less than 2%) of these burrows were resting burrows^3^. Resident pangolins use the resting burrows within their home range in turn, but infrequently return to the same burrow several days in a row. In the wet (summer) season, they use the same resting burrow continuously for an average of 1.5 days, while in the dry (winter) season the average is 3.9 days^3^. Also, unlike foraging burrows, which are created mainly in the dry season, are rarely revisited, and eventually collapse or fill up with earth, these resting burrows are permanent, being repeatedly used and shared, but non-simultaneously by different individuals, and they are routinely maintained by the users^3^.

Due to the difficulty of locating pangolins in the wild, knowledge concerning their reproductive biology, in comparison with phylogenetically closely-related carnivorous species^6^, is extremely limited^7,8^. Thus, almost all the present knowledge has come from captive observations. Records from captivity have shown that estrus and mating principally occur in the spring and summer (Feb.-Jul.) and the gestation period typically lasts six to seven months^4,9,10^. However, longer gestation length, based on the observation of the duration between mating behavior and parturition in captivity, has also been reported to range from 10 to more than 12 months in some cases^11^. Parturition in Chinese pangolins occurs seasonally between September and March, while there is a clear peak birth season from Dec. to Jan.^10^ (K. J.-C. Pei unpub. data).

We recently reported the first case of the growth and behavioral development of an infant pangolin raised by a radio-tagged young female (LF28) in their natural habitat^8^. It was found that pangolins exhibit maternal care. As infant pangolins require intensive maternal care during the nursing period^12^, knowledge of the reproductive behaviors of females during the nursing period not only has biological significance but is also crucial for the conservation of this critical endangered species. According to Sun et al.^8^, the infant pangolin was kept in the same nursing burrow after birth for approximately 4 weeks, before LF28 moved it to another burrow to continue nursing. During the whole nursing period, this mother–offspring pair moved to several resting burrows before the infant eventually left the mother at approximately 5 months old^8^.

In this article, we present further reports of resting burrow use patterns of the female pangolin LF28 and multiple male visitations during the nursing period based on intensive monitoring by radio-tracking and camera trapping from late 2012 to mid 2016. Two occasions of births, in 2014 and 2015, respectively, were also observed during the monitoring period. The body mass of this female pangolin was also recorded periodically. To our knowledge this is the first study to report such reproductive behavioral findings for any pangolin species, which filled in essential knowledge gaps for the conservation of these highly endangered species. Moreover, we also demonstrated the importance of using modern technology in furthering our understanding of the biology of a poorly studied species.

## Materials and methods

### Ethics statement

Ethics approval was granted by the Laboratory Animal Center, National Pingtung University of Science and Technology (NPUST). Pangolins were live captured for radio-tracking with permission granted by the Taiwan Forestry Bureau (permit numbers 1011701139, 1031700176, and 1050143346) as required by the Wildlife Conservation Act, 2013. All clinical examinations were carried out by experienced veterinarians following procedures described in Khatri-Chhetri et al.^13^ and all methods were carried out in accordance with the ARRIVE guidelines and regulations. This research is part of the “Pangolin biology and ecology project”, a long-term study conducted by the Institute of Wildlife Conservation, NPUST since 2009.

### Study area

This study was conducted in Taitung County, eastern Taiwan (22°54′N, 121°09′E), located at the southern end of the Coastal Mountain Range with an elevation range between 100 and 200 m a.s.l. Due to a long history of human encroachment, there is no primary forest in the study area, and the landscape is highly fragmented, which is interspersed with secondary forest, managed tree and bamboo plantations, grassland, agricultural land and a very low density of human settlements (< 7 people/km^2^). It is one of the few areas in Taiwan where a stable population of pangolins can be found^14^.

The climate of the study area consists of tropical weather, with hot, rainy months from April to November and colder, drier months from December to March. The average annual rainfall is about 1900 mm. Other large mammals that inhabit this area include the Formosan macaque *Macaca cyclopis*, wild pig *Sus scrofa*, Formosan serow *Naemorhedus swinhoei*, Reeves's muntjac *Muntiacus reevesi*, masked palm civet *Paguma larvata*, ferret-badger *Melogale moschata*, and crab-eating mongoose *Herpestes urva*.

### Radio tracking

Researchers encountered the female pangolin LF28 in the field on Oct. 4, 2012. She was brought to the Pingtung Rescue Center for Endangered Wild Animals (PTRC), NPUST, for clinical inspection and sample collection, including body weight and length measurements, blood sample collection for biochemical analyses, abdominal ultrasonography to check for pregnancy, microchip implantation and VHF radio-transmitter attachment. The transmitter was attached on a scale of the pangolin’s tail near the hip following the protocol suggested by Sun et al.^15^. Since LF28 was still growing when we started tracking in late 2012, two models of radio transmitter (Model R2020 12 g and R2030 24 g, Advanced Telemetry Systems, Inc, Isanti, MN, USA) with active mode of 16 h on/ 8 h off had to be used in the present study.

On Oct. 4, 2012, LF28 had a body weight of 1.85 kg and total body length of 60.2 cm, and was estimated to be 9–10 months old. Her birth month was estimated to be either Dec. 2011 or Jan. 2012 (i.e., 2011/2012 birth season). After LF28 was released where she was encountered, her subsequent locations were determined using a TR4 telemetry receiver (Telonics, Inc., 932 E. Impala Avenue, Mesa, AZ, 85204-6699 USA) with a directional antenna (RA-2AK or RA-23K; Telonics, Inc.). Triangulation was normally undertaken once a day for 7 consecutive days, and for 2 separate weeks per month. Other than tracking her in the nighttime when she was active, we also tracked the radio signals as frequently as possible in the daytime to locate her resting burrows.

The radio signal was un-detectable on Jan. 22, 2013, and LF28 was unable to be tracked until 1 year later, when she was approximately 2 years old. On Jan. 10, 2014, LF28 was in sight again in her home range. She carried a non-functioning transmitter with a detached antenna, and her identity was confirmed by microchip scanning. LF28’s transmitter was replaced on the same day and she was tracked without issue till June 16, 2016. The lighter transmitter was replaced by the heavier one when LF28’ reached a body weight of 3500 g. Home range and core activity area of LF28 were calculated using Minimum Convex Polygons (MCP)^16^.

During the whole monitoring period, LF28 was recaptured and brought to the field station, except during the nursing period, from time to time to check the transmitter condition^15^, milking status, and to measure her body weight. LF28 was sent to PTRC for a detailed clinical examination and sample collection three more times, which were in Jan. 2014, Aug. 2015 and May 2016, respectively, as descripted above.

### Intensive monitoring of LF28 during the nursing period

Once parturition was detected intensive monitoring was initiated to follow LF28 even more closely. The radio-tracking frequency was further increased to almost every day. We also increased our monitoring effort on the resting burrow used by LF28 by installing camera traps (Bushnell Trophy Cam, Reconyx HC500 or Reconyx UltraFire) at 1 to 1.5 m in front of the burrow entrance. Once LF28 had moved to another burrow, which was detected by radio-tracking, we relocated the camera trap immediately to the new site. Other pangolins that visited the burrows were also recorded. As the camera traps were set in video mode, we were able to identify the gender of almost every pangolin that approached the burrow based on the appearance of the genitals.

## Results

**Figure 1:**
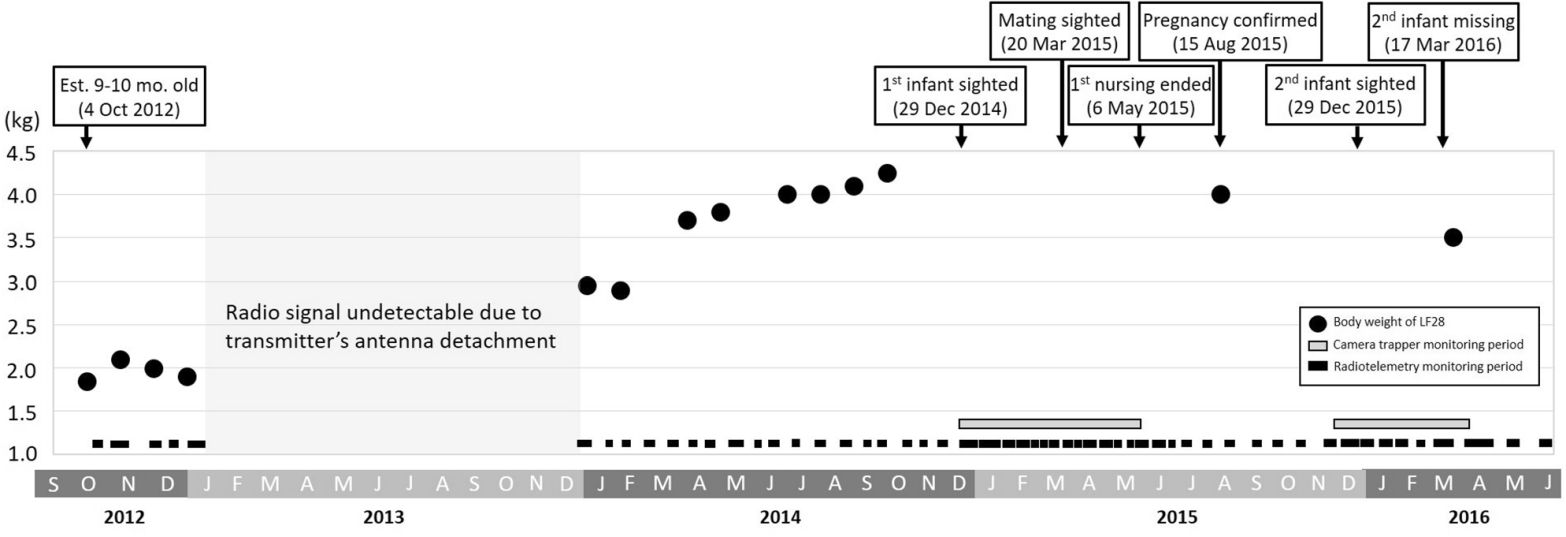
The tracking timeline, body mass, and reproduction records of a wild Chinese pangolin *Manis pentadactyla* (LF28) recorded from October 2012 to June 2016 in eastern Taiwan.

LF28 was radio-tracked regularly from Oct. 4, 2012 to June 16, 2016, except between Jan. 21, 2013 and Jan. 10, 2014 when the radio-transmitter was not functioning properly (Fig. 1). Two intensive monitoring sessions were undertaken: (1) from Dec. 10, 2014 to May 20, 2015, and (2) from Nov. 25, 2015 to Mar. 17, 2016 (Fig. 1).

### LF28’s body weight growth and two cases of birth

The body weight of LF28 increased from 1.85 kg in Oct. 4, 2012 to 4.00 kg in July 3, 2014 and then leveled off (Fig. 1). No sign of pregnancy or lactation was detected in LF28 during both 2012/2013 and 2013/2014 birth seasons. LF28 was estimated to have given birth to her first offspring on Dec. 1, 2014^8^, when she was roughly 3-years old (Fig. 1). The nursing period ended on May 6, 2015 when LF28 left the nursing burrow alone and after this date LF28 was not recorded with the offspring. The total length of this nursing period was 157 days.

**Figure 2:**
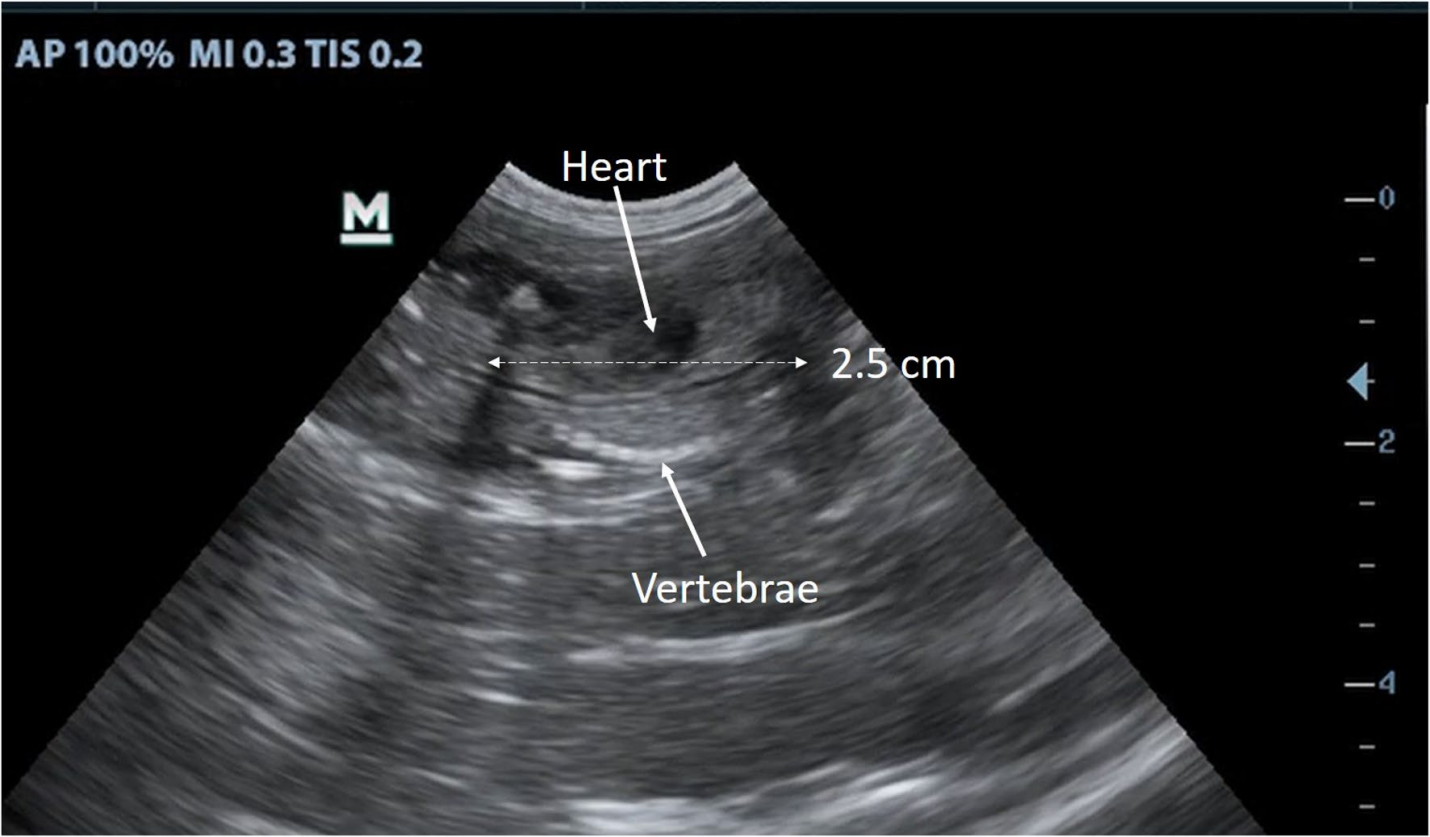
Ultrasonic image and embryo measurements of a female Chinese pangolin *Manis pentadactyla* (LF28) on 15 August 2015. LF28 then gave birth on 8 December 2015.

On August 15, 2015, approximately 3 months after the maternal separation of the first offspring LF28 was found to be pregnant again during abdominal ultrasonography, the crown-rump length of the embryo was 25 mm with heartbeat and vertebrae observed (Fig. 2). Our intensive monitoring resumed on Nov. 25, 2015 due to the expectation of delivery of the second offspring.

**Figure 3:**
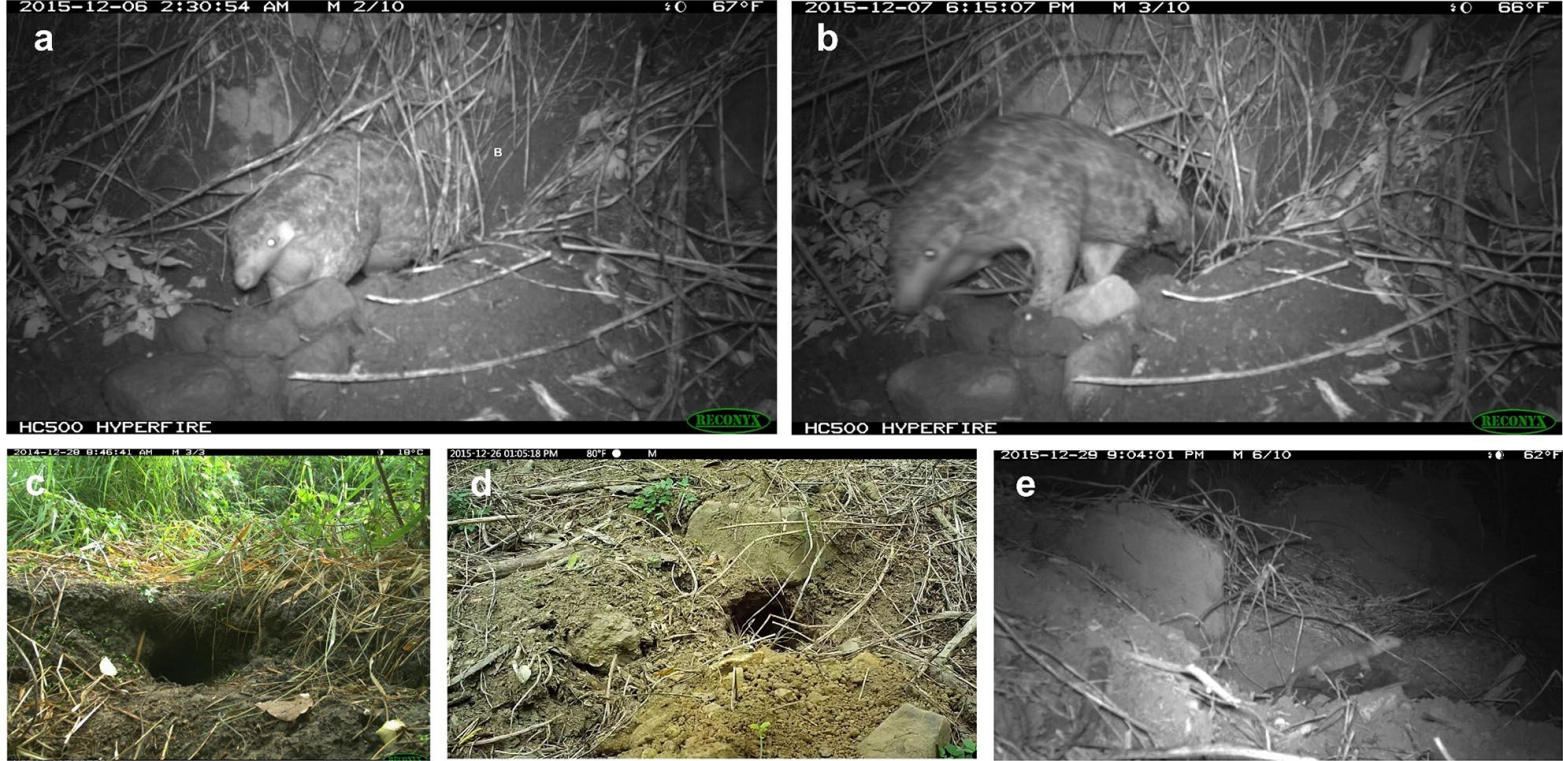
Determination of date of delivery and the parturition burrows used by a female Chinese pangolin *Manis pentadactyla* (code: LF28)*.* LF28 was still pregnant on Dec. 6, 2015 (**a**), and already delivered on Dec. 7 (**b**). The parturition burrows for the first birth (**c**) and the second birth (**d**). LF28 carried the second infant pangolin on Dec. 29, 2015 (**e**).

Photos taken at midnight on Dec. 6, 2015 indicated LF28 was still pregnant (Fig. 3a), however photos taken 16 h later revealed she had delivered her offspring (Fig. 3b). Therefore, the parturition of this second offspring took place from Dec. 6 ~ 7, 2015, which was almost exactly 1 year after the previous birth.

The second infant was captured for the first time on camera on Dec. 29, 2015, when it was 3 weeks old and carried by LF28 to relocate to another nursing burrow (Fig. 3e). However, despite LF28 being recorded 22 times during the next 2.5 months, this infant was never sighted again. We therefore believed the second birth was un-successful and intensive monitoring concluded on Mar. 17, 2016. Furthermore, the radio signal of LF28 was permanently lost due to unknown reasons on June 16, 2016 (Fig. 1).

### Resting burrow usages during the nursing period

**Figure 4:**
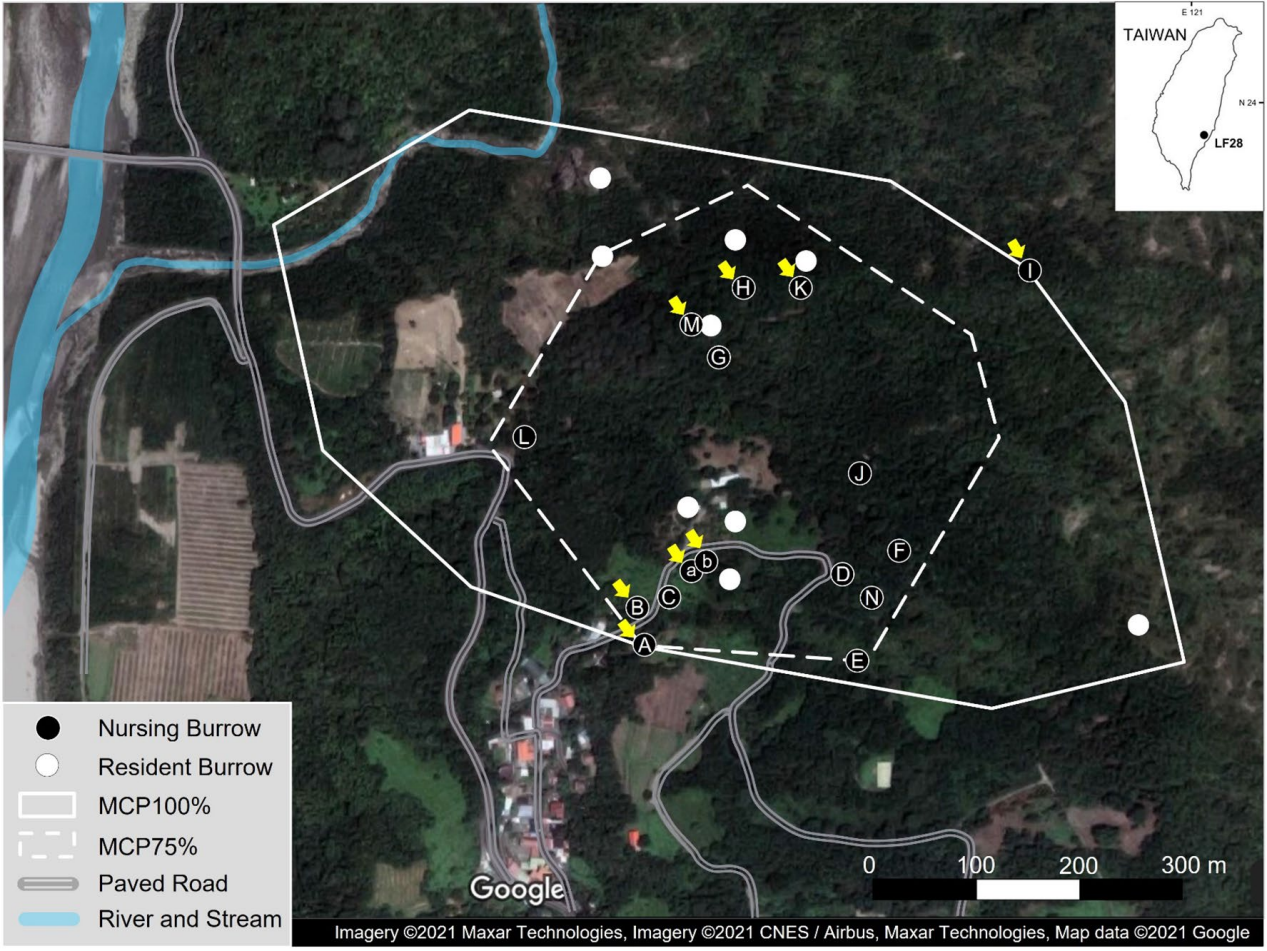
Study area showing the landscape, burrow sites and home range (minimum convex polygon, MCP), of a female Chinese pangolin (*Manis pentadactyla*) LF28 monitored from 2012 to 2016. The nursing burrows (black circle dot) were listed alphabetically in order of usage during the entire nursing period for her first offspring. The arrows by the natal burrows indicated the burrows where the male pangolins visited.

A total of 147 locations, including 122 nighttime locations and 25 resting burrows, were obtained by radio-tracking during the entire period of this study. The home range (MCP100) and core area (MCP75) sizes were 34.0 ha and 14.9 ha, respectively (Fig. 4). The core area of LF28 was located toward the southern edge of the home range, and as close as only 50 m to human settlements (Fig. 4). Among the 25 resting burrows, at least 16 (64%) were used as nursing burrows, including parturition, by LF28 during the two nursing periods. Fifteen of these 16 nursing burrows were located in the core area (Fig. 4). The majority of the nursing burrows were located in secondary forest (9), and the other habitats included bamboo plantation (3), grassland (2) and farmland (2). Interestingly, one of the farmland burrows was found under a seriously damaged concrete floor in a small, abandoned aviary.

The duration that LF28 used the same nursing burrow ranged from 1 day to over 35 days. The longest durations occurred when the parturitions took place (> 32 days and > 35 days respectively), and these two parturition burrows were located in bamboo plantation and farmland habitat, respectively (Fig. 3c,d). LF28 was frequently observed to pull hay into or out of the burrows during the parturition and nursing period. The hay-pulling behavior lasted as long as 46 min. LF28 left the nursing burrow every or every other day for foraging during the nursing period.

**Figure 5:**
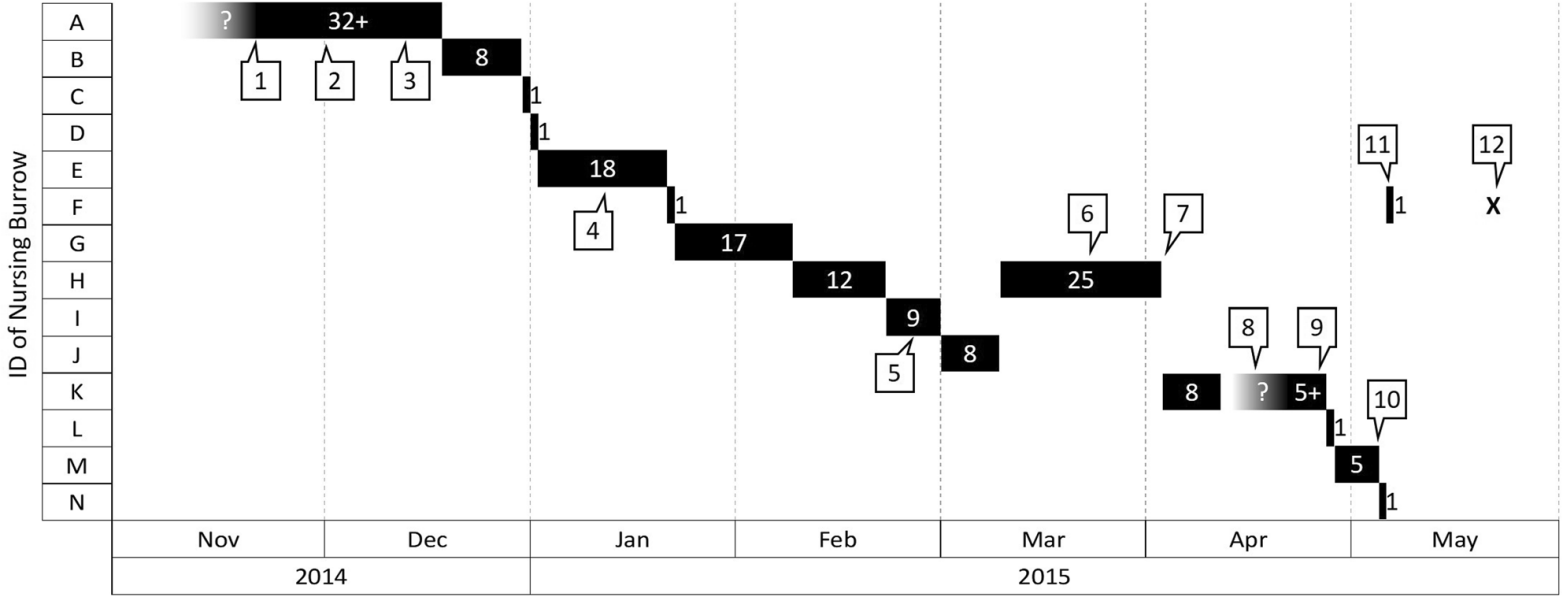
The nursing burrows used by a female Chinese pangolin *Manis pentadactyla* (LF28) during the nursing period. The nursing burrow ID and the order of usage refer to Fig. 4. The white numbers in the black blocks refer to the days of burrow use. On Nov. 21: starting date of radio-tracking session (No. 1). On Dec 1: parturition date of the first offspring (No. 2). On Dec. 13: one adult male entered this burrow at 02:28AM and excavated the soil and pushed the soil out of the burrow for 30 min. and then left (No. 3). On Jan. 9: one sub-adult pangolin (sex was unidentified) entered the burrow at 22:46PM and left within 1 min (No. 4). On Feb. 26: one marked adult male (LM15) entered the burrow and left within 4 min (C). LM15 was captured and scale-marked in October 2011 (No. 5). On March 20: one male entered the burrow at 00:44AM and following LF28 came out at 01:38 AM. The male then exhibited courtship and mating behaviors toward LF28 next to the burrow’s entrance. LF28 returned to the burrow alone at 01:44 AM (No. 6). On April 1: one adult male entered the burrow at 00:23AM and left 10 min later (No. 7). Apr. 11–21: LF28 carried the offspring and left burrow K, but her radio signal could not be tracked despite tracking efforts by the researchers. Intensive monitoring was not successive until LF28 was found in Burrow K again on 22 April (No. 8). On Apr. 26: one adult male entered the burrow at 00:34AM and left 6 min later (No. 9). On May 3: one adult male entered the burrow at 21:09PM and left 3 min later (No. 10). On May 6: LF28 was recorded leaving the offspring and was never sighted with the offspring afterwards (No. 11). On March 17: intensive monitoring stopped; radio-tracking continued every other week (No. 12).

LF28 used at least 14 resting burrows a total of 17 times during the first nursing period, with burrow F, H and K used twice (Fig. 5). On April 11, 2015, LF28 left burrow K with her offspring at 00:57, but her radio signal could not be tracked despite the efforts by the researchers during the following days. Intensive monitoring was therefore suspended until LF28 was observed back in burrow K again on April 22 (Fig. 5).

### Presence of other pangolins and small carnivores

**Figure 6:**
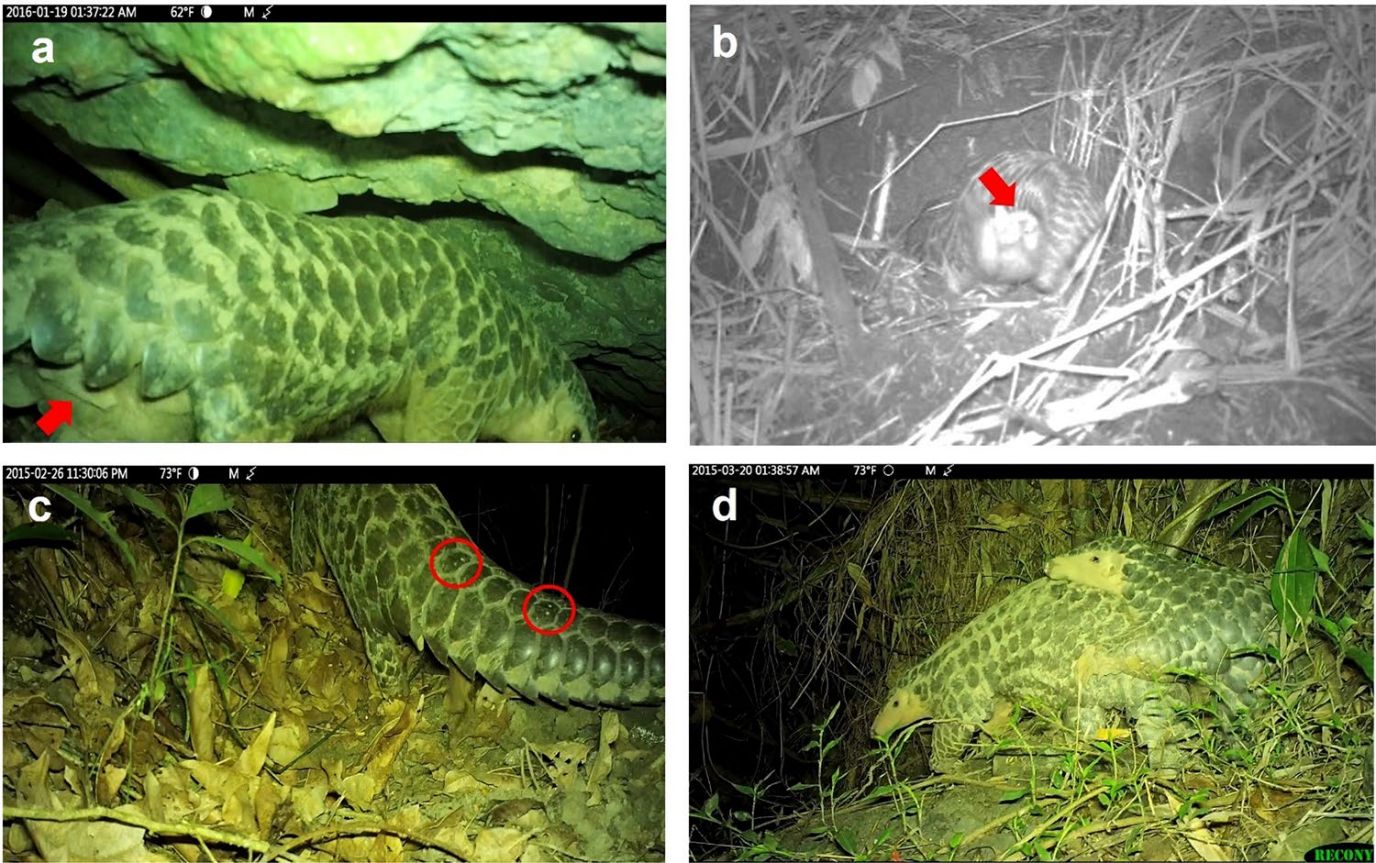
Male Chinese pangolins *Manis pentadactyla* visit the nursing burrows of a female pangolin. Two unknown individuals (**a**,**b**) were identified as males by the genitals, and one known male LM15 was identified by the scale mark (**c**). The female mated with an unknown male on 20 March 2015. The female was nursing a 4-month-old infant pangolin at the time (**d**).

During our intensive monitoring we were also able to record other pangolins and carnivores that approached or even entered the nursing burrow when it was occupied by LF28 and the infant. There were seven cases where other pangolins approached and entered the burrow, which took place between mid-Dec 2014 and early May 2015 (Fig. 5). With the exception of one subadult whose sex could not be discriminated from the video footage, adult males were responsible for all other visits (Fig. 6a,b). The adult male that visited on Dec. 13, 2014, not only entered the burrow but also showed soil excavation behavior. Moreover, the adult male observed on Feb. 26, 2015 was identified as an individual (LM15) that was once radio tagged by a research team in Oct. 2011 (Fig. 6c). Therefore at least two different adult males were recorded visiting the burrows during this nursing period. Of these seven visitations, five lasted less than 10 min, whereas the other two lasted for 30 min and 1 h, respectively. Mating behavior was recorded outside of the nursing burrow between an unidentified adult male and LF28 on March 20, 2015 (Figs. 5, 6d), when the infant pangolin was close to 4 months old.

During the second nursing period of LF28, four visitations from unmarked adult males were observed. Two of these visits were recorded at the parturition burrow, which included one 2-min long event at 01:28 on Dec. 6, right before the parturition, and one event 7 days later at 18:38 on Dec. 13. During the latter event, the male exhibited soil excavating behavior. We were not able to confirm the duration of the other two visitations performed by unmarked adult males, on Jan. 16 and Jan. 19, 2016, respectively, at the second burrow that LF28 used.

In addition to pangolins, small carnivores approached the nursing burrow on five separate occasions during the nursing period of LF28, comprised of three visitations of crab-eating mongoose and two visitations of ferret-badgers. Among the five visitations, two resulted in a carnivore entering the burrow and leaving after 2 min, which were each performed by a crab-eating mongoose and ferret-badger, respectively.

## Discussion

Despite only focusing on one female Chinese pangolin, LF28, our study, to our knowledge, is the first to provide highly detailed records on the nursing behavior of this poorly studied but critically endangered species. During the entire tracking period, the body weight of LF28 increased from 2 kg at the age of 1 year to 3 kg at the age of 2 years, and LF28 reached her maximum body weight of 4 kg at the age of 3 years. Based on the uninterrupted monitoring between Dec 2014 and June 2016, LF28 gave birth to her first offspring when she was 3 years old and another one at the age of 4 years (Fig. 1). Both infants were born in early December, which were in accordance with the peak birth season of the species^10^. Our observations confirmed that the Chinese pangolin is a seasonal breeder in the wild, and they give birth once a year^10,11^. Also, they can give birth in consecutive years with a litter size of one^17^.

Other studies (n = 4) have found that the lightest weight, or youngest age, a female Chinese pangolin can give birth at the age of 2 years or weight of 3 kg^11,17,18^, which indicated that they can conceive at an age of 1–2 years. Therefore, the first birth of LF28, which took place when she was 3 years old, might suggest a delay in pregnancy or sex maturation. However, information concerning the average primipara age for this species is not available to date, more research, especially in the wild, is necessary.

Our results indicate that female Chinese pangolins will carry their offspring frequently from one nursing burrow to another during the entire nursing period. In the case of LF28, nursing burrows were only some of the resting burrows utilized and were predominantly located within the core area (MCP75) of her home range (Fig. 4), despite the close proximity to human settlements. This suggests that familiarity of the environment or food resource availability should be important considerations in nursing burrow selection.

Nursing burrows were normally used only once during the same nursing period, with durations varying from 1 day to more than 1 month (Fig. 5). This frequent relocation behavior should be important to avoid predation of the newborn. Our monitoring showed that small carnivores, such as ferret-badgers or crab-eating mongoose, will enter the nursing burrow, which may suggest they are searching for prey. Therefore, this could reflect a potential threat to the infant pangolin, especially when the mother is absent for foraging^8^.

Burrows where LF28 gave birth were not only used for the longest duration after birth, they were also used before parturition. Similar to our findings, a previous study reported that both males and females will collect and pull hay into the resting burrow in the wintertime^3^. Therefore, in addition to providing insulation, the hay could also serve as necessary bedding for the delivery and nursing of offspring. Other functions of hay that have been proposed include false barriers that can act as predator deterrent structures^19^.

Our records revealed at least two different adult male pangolins approaching and entering the nursing burrows multiple times throughout the nursing period. Most of these visits lasted only minutes, whereas a few lasted longer. During one long visit, in March, mating behavior was observed, therefore the occurrence of post-partum estrus, or even ovulation, may be likely for this species. In captivity, mating behavior was also observed between February and July^10,20^. Although there is no direct evidence yet, these adult male visits suggest that at least some of them were for mate-searching. It has been proposed that while mammalian females spend more energy on parental care, males often invest more energy towards seeking mates^21^. For solitary and fossorial species such as the pangolin, a male’s mate-finding tactics can be critical for mating success, especially due to the low population density^22,23^. Male pangolins most likely depend on olfactory cues to locate females in heat. In mammals, female chemical signals have important roles in sexual attraction and facilitating sexual receptivity^24–27^. Female Chinese pangolins tend to defecate close to the burrow during the nursing period (N.C.M. Sun unpubl. data), therefore, despite the frequent relocation behavior expressed by the mother, it was likely to generate sufficient olfactory information for male pangolins.

It is also possible that female pangolins will mate more than once, even with different males, during the same nursing period. Sun et al.^17^ have reported that certain female Chinese pangolins exhibited a lack of mate fidelity based on microsatellite marker assessments. Our observation provides additional support for this phenomenon. Multiple mating with the same or different males has been observed in several solitary carnivores^28–31^. For males, frequent pre-copulatory encounters with females may offer advantages that increase opportunities for mating compared to males that are less familiar with females^32,33^. Hypotheses concerning the advantages of females exhibiting promiscuity have also been widely proposed, including direct benefits (e.g., stimulation of reproduction, fertilization assurance, mate retention etc.) and genetic benefits (e.g., choice of paternity, sperm competition, inbreeding avoidance etc.)^34,35^.

Interestingly, during two separate visitations adult males exhibited excavation behavior, and both events took place shortly after parturition. This excavation behavior at a parturition burrow has never been reported before for male pangolins, therefore, further research is needed to better understand the role male pangolins play in parental care.

The fetus of LF28’s second offspring detected in the ultrasonographic image in Aug. 15 provided additional information on the gestation length of the species. Following the fetal and extra-fetal structure development of small-sized (3–8 kg) dogs described in Luvoni and Grioni^36^ and Kim and Son^37^, we estimated the gestation period of this fetus may have lasted 30–40 days or less. The implantation of the blastocyst, therefore, most likely occurred in early July. This infant pangolin was born on Dec. 8 later that year, and the gestation length was estimated to be around 150 days, which was shorter than previous reports^4,9,10^. This was the first estimation of gestation length of the Chinese pangolin based on physiological evidence under natural conditions.

Our findings of the gestation period, which took place later in the year (July–December), coupled together with the occurrence of post-partum estrus and mating earlier in the year (December–May), suggests that delayed implantation likely takes place in this species, as proposed by Chin et al.^11^. This also explains why there was such an extensive variation in the gestation length, from 180 to more than 372 days, determined based on the observation of mating behavior and parturition in captivity^10,11,18^. More studies on the reproductive physiology for this species are necessary.

Lastly, the present study also demonstrated that the difficulties associated with researching the life history and behaviors of the elusive pangolin could be alleviated with the use of technologies (e.g., camera trapping, radio tracking, etc.). This is especially true for non-migratory fossorial species if one has an appropriate knowledge of their home range or residential environment. There are more and more new technologies and devices that have been developed and applied to wildlife research in the field, which should greatly improve our understanding and promote conservation efforts of endangered species such as the pangolin.
